# Supplementary material for: Prescriptive analytics for reducing 30-day hospital readmissions after general surgery
Source: PLoS One. 2020 Sep 9;15(9):e0238118. doi: 10.1371/journal.pone.0238118 (PMC7480861; doi:10.1371/journal.pone.0238118)
Supplement: S1 Appendix — (DOCX) [file pone.0238118.s001.docx]

**APPENDIX S1**

**Prescriptive Support Vector Machines (P-SVM)**

**Predictions**. Prescriptive Support Vector Machines (P-SVM) is a prescriptive method that is based on Sparse Linear SVM (SLSVM). To formulate the SLSVM problem, let $(x_{i}^{+},y_{i}^{+}), i=1,\ldots,N^{+}$, denote the ($D+1)-$dimensional positive samples, where $x_{i}^{+}$is the $D-$dimensional vector of variables for sample $i$ and $y_{i}^{+}=1$ the class label. Similarly, $(x_{j}^{-},y_{j}^{-}), j=1,\ldots,N^{-},$denote the negative samples (patients who are not re-admitted within 30 days) with $y_{i}^{-}=-1$. Let $\left( \boldsymbol{\beta},\beta_{0} \right)$be the vector orthogonal to the SVM hyperplane. Let also $M$be a parameter controlling the level of sparsity. Training a classifier amounts to selecting $\left( \boldsymbol{\beta},\beta_{0} \right)$so that the margin of the hyperplane is maximized:

$${min}_{\boldsymbol{\beta},\beta_{0}}\frac{1}{2}{\|\boldsymbol{\beta}\|}^{2}+\lambda^{+}\sum_{i=1}^{N^{+}} \xi_{i}+\lambda^{-}\sum_{j=1}^{N^{-}} \zeta_{j}$$

$$s.t. \sum_{d=1}^{D} \left| \beta_{d} \right|\leq M,$$

$$\xi_{i}\geq1-y_{i}^{+}\beta_{0}-\sum_{d=1}^{D} y_{i}^{+}\beta_{d}x_{i,d}^{+}, \forall i=1,\ldots,N^{+},$$

$$\zeta_{j}\geq1-y_{j}^{-}\beta_{0}-\sum_{d=1}^{D} y_{j}^{-}\beta_{d}x_{j,d}^{-}, \forall j=1,\ldots,N^{-},$$

$\xi_{i},\zeta_{j}\geq0, \forall i=1,\ldots,N^{+}, j=1,\ldots,N^{-}$.

This is a convex quadratic optimization problem and can be solved very efficiently for large training sets involving thousands of patients. Let $\left( \boldsymbol{\beta},\beta_{0} \right)$be an optimal solution of the problem above. Then, for a patient represented with a vector of variables $\boldsymbol{x}$ we compute $\beta_{0}+\sum_{d=1}^{D} x_{d}\beta_{d}$ and compare it with some threshold. If this value is above the threshold, we predict that the patient will be re-admitted. Otherwise, we predict it will not. The threshold can be set using cross-validation given a desirable false positive probability.

**Prescriptions**. Fixing the hyperplane $\left( \boldsymbol{\beta},\beta_{0} \right),$ we next consider each patient $i$ in the training set and seek to optimize the value of “actionable” variables $x_{i,d}^{+}$, for $d∊ Ϲ$, where $Ϲ$ is the index set of actionable variables, so as to “flip” the patient to the negative side of the hyperplane. To that end, we solve the following convex optimization problem. The objective is a linear combination of a penalty for not placing the patient on the negative side of the hyperplane and a penalty for altering the values of the variables characterizing the patient:

$${min}_{\xi, \boldsymbol{y}} \lambda\xi+{|\left| \boldsymbol{y}-\boldsymbol{x}_{i}^{+} \right||}_{p}^{p}$$

$$s.t. \xi-1\geq\beta_{0}+\sum_{d=1}^{D} \beta_{d}y_{d},$$

$$y_{d}=x_{i,d}^{+}, \forall d\notinϹ,$$

$$\xi\geq0,$$

$L_{d}{\leq y}_{d}\leq U_{d}$, $\forall d\inϹ,$

where ${||.||}_{p}$denotes the p-norm, $L_{d}$and $U_{d}$ are lower and upper bounds on the actionable variables, and $\lambda$ is a parameter trading-off the two penalty terms in the objective. The parameter $\lambda$ can be determined by validating the performance of the prescription determined by the above formulation in a validation dataset. After we fix $\lambda,$ we can solve the above problem for each patient in the test set to determine the optimal value of the actionable variables.
