# Supplementary material for: Prescriptive analytics for reducing 30-day hospital readmissions after general surgery
Source: PLoS One. 2020 Sep 9;15(9):e0238118. doi: 10.1371/journal.pone.0238118 (PMC7480861; doi:10.1371/journal.pone.0238118)
Supplement: S2 Appendix — (DOCX) [file pone.0238118.s002.docx]

**APPENDIX S2**

**Optimal Prescriptive Trees (OPT)**

We motivate and present the Optimal Prescription Tree (OPT) algorithm that trains prescriptive trees to directly minimize the personalization risk.

## **Personalization Risk.** We consider data such that $Y$ is the outcome for each patient, $T$ the choice of treatment and $\boldsymbol{X}$ the feature vector. First, we establish the convention that the smaller the outcome the better. Hence, we would like to minimize the expected outcome $E[Y(\tau\left( \boldsymbol{X} \right))]$ with respect to a prescriptive rule $\tau\left( \boldsymbol{X} \right)$. For a given dataset, the discretization of the expected value is thus:

$$R\left( \tau\right)=\sum_{i=1}^{n} Y_{i} 1\left[ \tau\left( \boldsymbol{X}_{i} \right)=T_{i} \right],$$

where $1\left[ \cdot\right]$ denotes the indicator function. We call $R\left( \tau\right)$ the *personalization risk*. However, in observational data we only observe the outcome for the treatment that was assigned to the patient in the data, and do not know what the outcome would be if a different treatment were prescribed. This leads to the corrected expression for the personalization risk:

$$R\left( \tau\right)=\sum_{i=1}^{n} \left( Y_{i} 1\left[ \tau\left( \boldsymbol{X}_{i} \right)=T_{i} \right]+\sum_{t\neq T_{i}} \hat{Y}_{i}(t) 1\left[ \tau\left( X_{i} \right)=t \right] \right),$$

where $\hat{Y}_{i}(t)$ denotes the unknown counterfactual outcome that would have been observed if patient $i$ were to be assigned treatment $t$.

Then, to further control for accuracy, we account for the quality of the counterfactual estimates. However, since we only know the true value of the outcome for one particular counterfactual, we will only control the quality of those. This leads to the squared loss term:

$$\sum_{i=1}^{n} \left( Y_{i}-\hat{Y}_{i}\left( T_{i} \right) \right)^{2},$$

where $T_{i}$ is the treatment corresponding to the point $(\boldsymbol{X}_{i},Y_{i})$ in the data. Forming the linear combinations of these two terms, we obtain the final objective:

$$R\left( \tau\right)=\mu\sum_{i=1}^{n} \left( Y_{i} 1\left[ \tau\left( \boldsymbol{X}_{i} \right)=T_{i} \right]+\sum_{t\neq T_{i}} \hat{Y}_{i}(t) 1\left[ \tau\left( \boldsymbol{X}_{i} \right)=t \right] \right)+\left( 1-\mu\right)\sum_{i=1}^{n} \left( Y_{i}-\hat{Y}_{i}\left( T_{i} \right) \right)^{2},$$

where$\mu$ is the *prescription factor*, a hyperparameter that controls the trade-off between the prescription error and the prediction error. Minimizing this function above over $\tau$ is the basis of Optimal Prescriptive Trees.

## **Prescription Predictions.** To minimize over $\tau$, we seek a decision rule that takes the form of a prescriptive tree, that is, a decision tree that in each leaf prescribes a common treatment for all samples. Our approach is to estimate the counterfactual outcomes using this prescriptive tree during the training process, and therefore jointly optimize the counterfactual estimation and minimization of personalization risk.

Observe that a decision tree divides the training data into clusters where the samples are similar. We propose using these clusters as the basis for our counterfactual estimation. More concretely, we will estimate the counterfactual $\hat{Y}_{i}(t)$ using the outcomes $Y_{j}$ for all samples $j$ with $T_{j} = t$ that fall into the same leaf of the tree as sample $i$. An immediate method for estimation is to simply use the mean outcome of the relevant samples in this cluster, giving the following expression for $\hat{Y}_{i}(t)$:

$$\hat{Y}_{i}\left( t \right)=\frac{1}{|\left\{ j:\boldsymbol{X}_{j}\in\Lambda_{l\left( i \right)}, T_{j}=t \right\}|}\sum_{\{j:\boldsymbol{X}_{j}\in\Lambda_{l\left( i \right)}\}} Y_{j},$$

where $\Lambda_{l\left( i \right)}$ represents the leaf of the prescription tree that $\boldsymbol{X}_{i}$ falls into. Then, using this expression, we want to find a prescriptive tree $\tau$ such that it solves the following problem:

$$R\left( \tau\right)=\mu\sum_{i=1}^{n} \left( Y_{i} 1\left[ \tau\left( \boldsymbol{X}_{i} \right)=T_{i} \right]+\sum_{t\neq T_{i}} \frac{1}{|\left\{ j:\boldsymbol{X}_{j}\in\Lambda_{l\left( i \right)}, T_{j}=t \right\}|}\sum_{\{j:X_{j}\in\Lambda_{l\left( i \right)}\}} Y_{j} 1\left[ \tau\left( \boldsymbol{X}_{i} \right)=t \right] \right)+\left( 1-\mu\right)\sum_{i=1}^{n} \left( Y_{i}-\frac{1}{|\left\{ j:\boldsymbol{X}_{j}\in\Lambda_{l\left( i \right)}, T_{j}=T_{i} \right\}|}\sum_{\{j:\boldsymbol{X}_{j}\in\Lambda_{l\left( i \right)}\}} Y_{j} \right)^{2}.$$

We then use local-search methods to optimize the splits of the prescriptive trees over this objective, with each one starting from different random splits. Specifically, we initialize many prescriptive trees with random splits, and iteratively apply the following steps:

1. We randomly select a tree $\tau_{i}$.
2. We randomly select a node $n_{ij}$ within the selected tree $\tau_{i}$.
3. We optimize the split at node $n_{ij}$ keeping all other splits constant by minimizing the function above.
4. We return to Step 1 and repeat.

The process is completed when all nodes are individually optimized. Then, the best tree is selected through validation. The hyperparameter $\mu$ is also chosen through the validation set.
