# Supplementary material for: Prescriptive analytics for reducing 30-day hospital readmissions after general surgery
Source: PLoS One. 2020 Sep 9;15(9):e0238118. doi: 10.1371/journal.pone.0238118 (PMC7480861; doi:10.1371/journal.pone.0238118)
Supplement: S3 Appendix — (DOCX) [file pone.0238118.s003.docx]

**APPENDIX S3**

To illustrate the behavior of our models under a subgroup analysis, we selected the largest subgroup, general surgeries, and retrained our models using only these surgeries. We further utilized the same splitting scheme for train/validation/test, and ran each model 3 times across 3 random splits. The average and standard deviation of the results across these 3 runs are listed in Table S1.

**Table S1. Performance of predictive models on the subgroup of general surgeries.**

|  | **POST-op** |  | **PRE-op** |  |
| --- | --- | --- | --- | --- |
| **Methods** | **Avg.** | **Std.** | **Avg.** | **Std.** |
| **L2LR** | 82.91% | 0.13% | 72.42% | 0.20% |
| **SLSVM** | 82.89% | 0.10% | 72.38% | 0.18% |
| **RF** | 84.10% | 0.12% | 72.91% | 0.28% |
| **GBM** | 85.21% | 0.21% | 73.71% | 0.34% |
| **NN** | 81.90% | 0.25% | 72.56% | 0.41% |
