# Supplementary material for: Prescriptive analytics for reducing 30-day hospital readmissions after general surgery
Source: PLoS One. 2020 Sep 9;15(9):e0238118. doi: 10.1371/journal.pone.0238118 (PMC7480861; doi:10.1371/journal.pone.0238118)
Supplement: S4 Appendix — (DOCX) [file pone.0238118.s004.docx]

**APPENDIX S4**

Table S2 reports the average specificity of our models across the three splits using the threshold such that the predicted readmission rate equals the ground truth readmission rate in the training dataset.

**Table S2. Average specificity of the predictive models.**

| **Methods** | **PRE-op specificity** | **POST-op specificity** |
| --- | --- | --- |
| **L2LR** | 95.20% | 96.71% |
| **RF** | 95.17% | 96.85% |
| **SLSVM** | 95.19% | 96.60% |
| **GBM** | 95.30% | 97.01% |
| **NN** | 94.89% | 96.80% |
