# Supplementary material for: Prescriptive analytics for reducing 30-day hospital readmissions after general surgery
Source: PLoS One. 2020 Sep 9;15(9):e0238118. doi: 10.1371/journal.pone.0238118 (PMC7480861; doi:10.1371/journal.pone.0238118)
Supplement: Attachment — Submitted filename: PLOS HCT - Response to Reviewers.docx [file pone.0238118.s005.docx]

**Response to the Academic Editor**

Dear Dr. Chun Chieh Yeh:

Thank you for the opportunity to revise our manuscript. We have updated the paper to address all of the comments raised by you and the reviewers. Below we address the comments you have raised. Please see our detailed responses to the reviewers for how we have updated the paper with respect to each comment.

*1. Blood transfusion is well known in association with post-operative recurrent cancer in oncologic surgeries. I think the long-term oncologic outcomes should be more important than short-term readmission rate. Could you use your dataset to study the influence of pre-operative blood transfusion in post-operative oncologic outcomes (e.g. disease free survival ) after oncologic surgeries (e.g. pancreas surgeries for pancreatic cancer or liver resection for liver cancer? or colon surgeries for colon cancer?)? OR, at least, you should address this in your discussion part.*

Thank you for your comment. We agree that long-term oncologic outcome is an important indicator of surgery success. Unfortunately, the NSQIP database does not track patients longer term and the readmission information is in fact the furthest information we have regarding the patient regarding the data. We have added such a statement into the discussion.

*2. Please revise your abstract to fit the format for publication (i.e., Introduction /Material and method/ Result/ Conclusion )*

Thank you for this comment. We have updated our abstract to fit the format for publication.

With best regards and on behalf of the authors,

Ioannis Ch. Paschalidis

Professor and Data Science Fellow,

Electrical & Computer, Systems, and Biomedical Engineering, and

Computing & Data Sciences,

Director, Center for Information and Systems Engineering,

T: (617) 353-0434, F: (617) 353-0190

[yannisp@bu.edu](mailto:yannisp@bu.edu)

<http://sites.bu.edu/paschalidis/>

**Response to Reviewer 1**

*1.Do you consider to separate different surgery type in analysis? or do subgroup analysis for different surgery type? (for example: abdominal surgery/non-abdominal surgery). I think it may increase AUC according to previous experience*

Thank you for the comment. The variables available to each model (under both post-op and pre-op variants) include the surgery specialty and primary CPT categories for the main procedure. Therefore, the surgery type information is available to the models in analysis.

To illustrate the behavior of our models under a subgroup analysis, we selected the largest subgroup, general surgeries, and retrained our models using only these surgeries. We further utilized the same splitting scheme for train/validation/test, and ran each model 3 times across 3 random splits. The average and standard deviation of the results across these 3 runs are as follows:

|  | **POST-op** |  | **PRE-op** |  |
| --- | --- | --- | --- | --- |
| **Methods** | **Avg.** | **Std.** | **Avg.** | **Std.** |
| **L2LR** | 82.91% | 0.13% | 72.42% | 0.20% |
| **SLSVM** | 82.89% | 0.10% | 72.38% | 0.18% |
| **RF** | 84.10% | 0.12% | 72.91% | 0.28% |
| **GBM** | 85.21% | 0.21% | 73.71% | 0.34% |
| **NN** | 81.90% | 0.25% | 72.56% | 0.41% |

Compared with the model results across all surgeries, we see that there is a 1-2% drop in AUC for the post-op case, and a smaller drop for the pre-op case. Thus, a specialized model that focuses on one class of surgeries does not outperform the general model across all surgery categories. We believe that this is due to favorable cross-learning across the different surgery classes.

These results are also included in the paper.

*2.Readmission is closely correlative with many factors in the previous hospitalization, especially post-operative outcome. Did you try to do predictive model using only post-operative variables?*

Thank you for this question. We agree that readmission is closely correlated with the post-operative outcome. We ran a predictive model using only post-operative variables (by excluding all variables included in the pre-op variables). Similar to the method outlined in the paper, we trained a model using a train/valid/test split of 60%/20%/20%, and ran each model across 3 independent random splits. The average and standard deviation of the results across these 3 runs are as follows:

|  | **only POST-op** | |
| --- | --- | --- |
| **Methods** | **Avg.** | **Std.** |
| **L2LR** | 81.02% | 0.09% |
| **SLSVM** | 80.79% | 0.15% |
| **RF** | 83.32% | 0.16% |
| **GBM** | 84.56% | 0.25% |
| **NN** | 83.79% | 0.14% |

We see that, as you pointed out, the models generate good and stable performance across 3 random splits of the data, indicating that the post-op results do significantly impact readmission. In our study, we focused on the pre-op model since we are looking to conduct a pre-op intervention that aims to reduce the readmission rate, and thus at the time of intervention, the post-op results are not yet known to us.

*3. In the aspect of clinical use, another possible application of this study is to decrease the length of hospital stay (previous hospitalization) in low risk patient. Do you evaluate the specificity(or false negative rate) of this model?*

Thank you for your comment. We agree that this is also a potential application of this study. The specificity of our models would depend on the cutoff chosen utilized to binarize the outcomes from our models. For example, we could select a low cutoff that has a high sensitivity but a low specificity, or vice versa. The following table reports the specificity of our models using the cutoff utilized in the paper, so that the resulting rate of readmission corresponds with the true data:

| **Methods** | **PRE-op specificity** | **POST-op specificity** |
| --- | --- | --- |
| **L2LR** | 95.20% | 96.71% |
| **RF** | 95.17% | 96.85% |
| **SLSVM** | 95.19% | 96.60% |
| **GBM** | 95.30% | 97.01% |
| **NN** | 94.89% | 96.80% |

The results show that our models show high specificity under this cutoff across the board, and out of all the results we identify as negative, over 95% of them are truly negative using the pre-op model, and about 97% are truly negative using the post-op model. The results are also included in the paper.

*4.How do you think about 87% and 74% AUC in AI study? Do you estimate if we want to achieve enough benefit(reduce readmission and save money), how higher AUC do we need?*

Thank you for your question. In this study, we have experimented with a wide class of machine learning methods (from logistics regression to gradient boosted trees and neural networks). Thus, the AUC results that we have achieved are likely representative of the maximum AUC achievable using currently available methods for these data.

As reported in the paper, with the current AUC, we estimate we can save more than $20 million annually if this system were to be implemented in the hospitals included in the NSQIP dataset. A further expansion of the features in the dataset would likely lead to higher predictive power and greater improvements.

*5. You did many machine learning methods in the study and also mention about “interpretable”. Please try to add more discussion about the machine learning methods and you result. It needs more integration (and explanation) of different machine learning methods, results and your clinical problems.*

Thank you for this comment. We added more explanation to our methodology in the methods section and expanded our discussion on the interpretability of the results.

**Response to Reviewer 2**

*1. The data showed a large national dataset of surgical patients with the goal of reducing 30-day readmissions. How can we interpret the relationship between blood transfusion and readmission, focussing on the listed complication such as septic shock, bleeding pneumonia, SSIs, Renal Insufficiency?*

Thank you for this comment. In our dataset, we observe a negative correlation with Hematocrit levels and various post-op complications, including septic shock (-0.06), bleeding pneumonia (-0.06), SSIs (-0.04), and renal insufficiency (-0.03). This negative correlation is also observed in the models after controlling for the other factors. As it is known, post-op complications greatly impact the chance of readmission (see response point 2 to Reviewer 1). Thus, one of the plausible interpretations is that pre-operative blood transfusion could reduce the probability of having a post-op complication, which in turn decreases the probability of being readmitted. For the example of septic shock, blood transfusion is well regarded as a standard therapy (see e.g. [1]), and thus this interpretation is supported by the medical literature.

**References**

[1] Chan, Y. L., Han, S. T., Li, C. H., Wu, C. C., & Chen, K. F. (2017). Transfusion of red blood cells to patients with sepsis. *International journal of molecular sciences*, *18*(9), 1946.
